# Sagittal balance parameters in achondroplasia

**DOI:** 10.1016/j.bas.2023.102670

**Published:** 2023-09-03

**Authors:** H. Cai, C. Omara, R. Castelein, C.L. Vleggeert-Lankamp

**Affiliations:** a Department of Neurosurgery, Leiden University Medical Centre, Leiden, the Netherlands; b Computational Neuroscience Outcome Center, Brigham and Woman's Hospital, Harvard Medical School, Boston, USA; c Department of Orthopedics, University Medical Centre Utrecht, the Netherlands; d Spaarne Gasthuis Haarlem/Hoofddorp, the Netherlands

**Keywords:** Achondroplasia, Lumbar spinal stenosis, Sagittal balance, Lumbar lordosis

## Abstract

**Introduction:**

Lumbar spinal stenosis (LSS) is the main problem for adult achondroplasia (Ach). Sagittal imbalance of the spine may play a role in LSS causing neurogenic claudication in Ach patients.

**Research question:**

The purpose of this study is to describe the sagittal balance parameters in Ach patients.

**Methods:**

A single-centre retrospective study of Ach patients that visited the Neurosurgery outpatient clinic of the Leiden University Medical Centre (LUMC) between 2019 and 2022 was performed. We defined sagittal imbalance by a C7 sagittal vertical axis (SVA) of more than 10 mm.

**Results:**

There were 13 patients with a spinal sagittal imbalance and 15 patients with a balanced spine. In both groups, the sacral slope (SS) was comparable (45.0° and 49.0°, p = 0.305), but exceeding the mean SS in non achondroplasts (38.0°). Lumbar lordosis (LL) was more pronounced in the balanced group (55.5° versus 41.7°, p = 0.019), and positively correlated to SS in contrast to the absence of a correlation in the imbalanced group. Thoracolumbar kyphosis (TLK) was increased comparably in both groups (19.6° and 24.6°), and far exceeding the TLK in non achondroplasts (circa 0°), and in both groups negatively correlated with the LL, although not enough to compensate for the smaller LL in the imbalanced group.

**Conclusion:**

Only if the LL compensates for both a larger SS and TLK, the Ach spine can maintain sagittal balance. An explanation for the current data can be the failure of the lumbar spine to give sufficient lordosis due to degenerative processes.

## Highlights

• The lumbar lordosis angle of the balanced spine is larger than that of the imbalanced patients.
• A significant positive correlation between the sacral slop and lumbar lordosis angle is found in balanced groups, indicating the compensation of the increased LL for the SS, which is larger than the reported values of the SS in non-achondroplasts.
• In patients with an imbalanced spine, there is a strong negative correlation between lumbar lordosis and thoracolumbar angle, denoting that the LL fails to compensate for thoracolumbar kyphosis. This correlation is likewise negative in the balanced patients, but with moderate strength.

## 1 Introduction

Achondroplasia (Ach) is the most common form of short limb dwarfism and it is caused by a gain-of-function mutation in Fibroblast Growth Factor Receptor 3 (Ornitz et al., 2017). The disorder has an estimated birth prevalence of about 4.6 per 100,000 births worldwide (Foreman et al., 2020). Ach is characterized by short stature, rhizomelic shortening of limbs, and frontal bossing. Although the length of the spinal column is comparable to that of non-achondroplasts, the aberrant anatomy frequently leads to clinical symptoms. Abnormal enchondral ossification results in untimely fusion of the pedicles to the vertebral body, thereby inducing a decreased anterior-posterior diameter of the spinal canal. Narrowing of the lumbar spinal canal predisposes compression of the cauda equina which is associated with symptoms of neurogenic claudication (Pauli, 2019). The prevalence of symptomatic lumbar spinal stenosis in Ach is approximately 10% and almost 80% of Ach patients have encountered an episode of neurogenic claudication by the time they have reached the sixth decade (Hunter et al., 1998). Narrowing of the canal in the thoracolumbar region is associated with symptoms of medullary compression, leading to clumsiness of the lower extremities, walking disability and urinary incontinence (Pauli, 2019).

It is hypothesized that the above-mentioned clinical symptoms in Ach are not only due to the decreased diameter of the spinal canal, but that the deviating sagittal balance parameters of the Ach spine contribute as well. The achondroplast spine is as well characterized by a ‘sacrum acutum’, a horizontally tilted body of the sacrum (Ando et al., 2021). In the sagittal balance parameters, this is characterized by a steep sacral slope (SS), that is approaching its final angle at the age of three (Ando et al., 2021; Abousamra et al., 2019). This steepness of the SS influences the values of the pelvic tilt (PT) and the pelvic incidence (PI), and may also affect the lumbar lordosis (LL).

Furthermore, the achondroplast spine frequently demonstrates a significant thoracolumbar kyphotic angle. In non achondroplasts, this angle is usually absent and in achondroplasia a TLK larger than 20° is considered to be abnormal (Mok et al., 2022). Pauli suggests that the very large head in combination with the characteristic hypotonia, particularly affecting the trunk, forces the thoracolumbar junction to bend if the child is in sitting position (Pauli et al., 1997). During childhood of the achondroplast patient, particular care should be given to avoid the sitting position in order to avoid the increase of TLK. This is not commonly done, and as a result, a thoracolumbar kyphotic angle larger than 10° still is present in ca 80% of adult achondroplasts (Khan et al., 2016). The presence of this angle adds to a sagittal imbalance and can cause clinical symptoms (Pauli et al., 1997; Hall, 1988).

The narrowing of the spinal canal, the horizontally tilted sacrum, and the thoracolumbar angle make the Ach vulnerable for developing a sagittal imbalanced spine. The objective of this study is to assess the dimensions of the sagittal balance parameters in achondroplasia and to explore their correlation.

## 2 Methods

### 2.1 Participants

Achondroplast patients that visited the Leiden University Medical Center (LUMC), Neurosurgery Clinic for people with small stature, with varying complaints concerning degeneration of the spinal column were asked to be subjected to a full spine anteroposterior and lateral radiograph. All patients seen at the clinic from 2019 to 2023 with a full spine radiograph were included in this study. Data were anonymized before evaluation.

### 2.2 Radiological data

Each enrolled patients received a full spine X-ray in a standing position with fingertips on their clavicles. The following measurements were taken from the radiographs: C7 sagittal vertical axis (SVA), thoracolumbar kyphotic angle (TLK), lumbar lordosis (LL), distal lumbar lordosis (DLL), sacral slope (SS), pelvic incidence (PI), pelvic tilt (PT) and adapted LL. C7 SVA was measured as the horizontal distance between a plumb line drawn from the center of C7 and the plumb line from the posterior-superior corner of the sacrum (Gelb et al., 1995). An SVA of less than 10 mm was defined as a ‘balanced spine’ and with an SVA of more than 10 mm as an ‘imbalanced spine’.

**Fig. 1:**
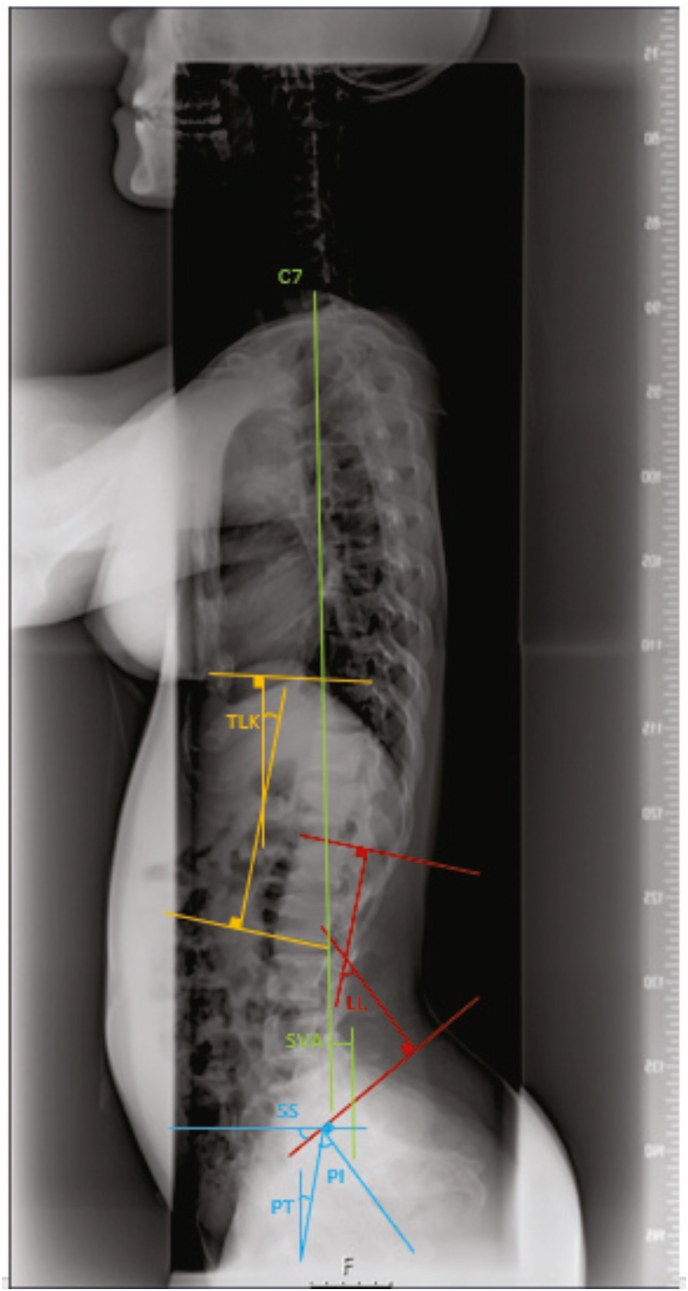
Measurement of sagittal balance parameters. All the assessment are performed on lateral spine radiographs. sagittal vertical axis (SVA) is measured as the horizontal distance between a plumb line from the center of C7 and the plumb line from the posterior-superior corner of the sacrum. Thoracolumbar kyphosis (TLK) is the Cobb angle between the superior endplate of T10 and the inferior endplate of L2. LL is measured as the Cobb angle between the superior endplate of L1 and the superior endplate of S1. Sacral slope (SS) is the angle between the superior endplate of S1 and the horizontal plane. Pelvic incidence (PI) is the angle between the perpendicular line to the sacral plate and the line connecting the midpoint of the sacral plate to the midpoint of the femoral heads. Pelvic tilt (PT) is the angle between the vertical axis arising from the midpoint of the femoral head and the line from the midpoint of the superior endplate of S1 to the center of the femoral head.

**Fig. 2:**
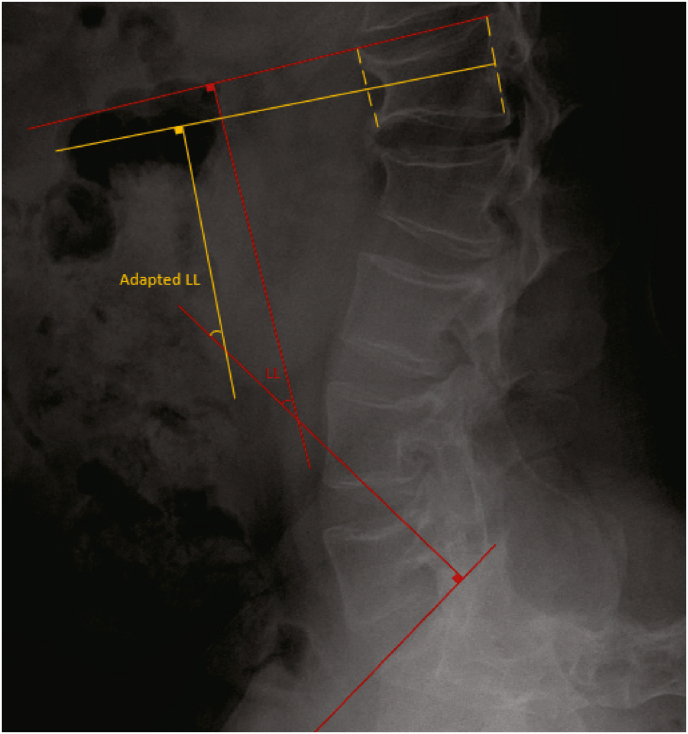
Adapted lumbar lordosis (LL) measurement to correct for wedging at L1 lumbar level. Adapted LL is measured as the Cobb angle between the superior endplate of S1 and the line between the midpoints of the anterior and posterior wall of the L1 lumbar vertebra.

TLK was measured as the Cobb angle between the superior endplate of T10 and inferior endplate of L2 (Margalit et al., 2018). A TLK of more than 20° was considered to be pathological (Mok et al., 2022). LL was measured as the Cobb angle between the superior endplate of L1 and the superior endplate of S1. DLL was the Cobb angle between the superior endplate of L4 and the superior endplate of S1. SS was measured as the angle between the horizontal line and the line along the superior endplate of S1. PI was measured as the angle between the perpendicular line from the sacral plate and the line connecting the midpoint of the sacral plate to the midpoint of the femoral heads. PT was measured as the angle between the vertical axis arising from the midpoint of the femoral head and the line running from the midpoint of superior endplate of S1 to the center of femoral head (Fig. 1) (Duval-Beaupère et al., 1992). Some patients demonstrated wedging of the L1 lumbar vertebra, which may affect the measuring of the Cobb angle. Therefore, additionally the adapted LL is measured, as the Cobb angle between superior endplate of S1 and the line drawn between the midpoints of the anterior and posterior wall of the L1 lumbar vertebra (Fig. 2).

### 2.3 Statistical analysis

Continuous values were reported with a mean and standard deviation (mean ± SD). Independent samples *t*-test or a Mann-Whitney *U* test were applied to compare the parameters between patients with a balanced and a imbalanced spine. Pairwise Pearson correlation was used to explore the correlation among spino-pelvic parameters. A coefficient value (R) greater than 0.60 was considered to be satisfactory (R = 0.41–0.60 [moderate], R = 0.61–0.80 [good] and R = 0.81–1.0 [strong]). All analyses were conducted using R software (version 4.2.1). Probability values < 0.05 were considered statistically significant.

## 3 Results

A total of 28 achondroplast patients with a lateral full spine radiograph were enrolled into the study. Of the 15 patients with a balanced sagittal spine, 6 patients demonstrated an SVA ranging from 0 to 10 mm, and 9 patients had a negative SVA value ranging from −0.7 to −31.7 mm. 13 patients had a imbalanced spine with an SVA ranging from 10.7 to 147 mm. The mean SVA of patients with a balanced spine was significantly lower than the SVA of patients with a imbalanced spine (– 7.3 ± 13.6 mm versus 65.0 ± 37.4 mm, p < 0.001). Mean age in both groups was not significantly different, but slightly higher in the imbalanced group (49.9 vs 39.9 yrs, p = 0.088; Table 1).

**Table 1:** Comparison of sagittal balance parameters between Ach patients with balanced and disbalanced spine.

	Balanced group	Disbalanced group	*p*-value	Roussoully = normal non-Ach
no. of patients	15	13		
Age	39.9 ±14.0	49.9±16.1	0.088	
female	9	5	1	
male	6	8		
SVA	−7.3 ± 13.6	65.0 ± 37.4	<0.001*	12.3
TLK	19.6 ± 20.7	24.6 ± 15.7	0.205	
LL	55.5 ± 15.7	41.7 ± 12.8	0.019*	55.9
SS	45.0 ± 10.6	49.0 ± 9.6	0.305	37.7
PT	18.9 ± 24.0	22.9 ± 17.4	0.645	15.1
PI	61.7 ± 31.2	71.7 ± 22.0	0.378	52.7
Adapted LL	59.7 ± 17.3	50.0 ± 12.0	0.100	
DLL	41.1 ± 8.7	43.3 ± 6.7	0.453	

*Statistically significant (p < 0.05).

There was no significant difference in SS between SVA balanced and imbalanced patients (45.0 ± 10.6° versus 49.0 ± 9.6°, p = 0.305). However, the SS in both groups was larger than the reported values of SS in non achondroplasts (37.7°) (Charles et al., 2022). Likewise, the PT and PI were comparable in the balanced and imbalanced patients, and both parameters were larger than in non Ach population (Table 1). There was also no significant difference in mean TLK in both groups (19.6 ± 20.7° versus 24.6 ± 15.7°, p = 0.205). The individual values though, had a tendency to demonstrate a higher TLK in the imbalanced spines. In the balanced spine, TLK ranged from 2.9° to 39.4° (19.6 ± 20.7°, with an outlier 81.0°), while it ranged from 6.0° to 57.6° (24.6 ± 15.7°) in the imbalanced patients. The lumbar lordosis angle is the parameter that is significantly different in the balanced and the imbalanced patients: the LL was larger in the balanced group (55.5 ± 15.7° versus 41.7 ± 12.8°, p = 0.019). However, the distal lumbar lordosis (between L4 and S1) was not significantly different between both groups.

10 patients demonstrated wedging of the L1 vertebra; 3 of these had an imbalanced spine and 7 had a balanced spine. If the LL was corrected for wedge shaped, lumbar lordosis was likewise larger in the balanced group, though not reaching significance (59.7 ± 17.3° versus 50.0 ± 12.0°, Table 1).

**Fig. 3:**
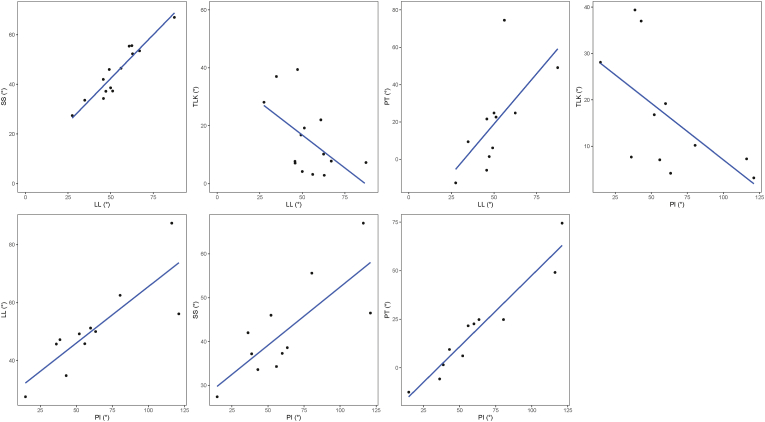
Graphical representation of the relationship between SS, LL, TLK, PT, and PI in achondroplast patients with a balanced spine. Positive correlations are identified between SS and LL, PT and LL, PI and LL, SS and PI, and PT and PI. Negative correlations exist between TLK and LL. TLK: thoracolumbar kyphosis, LL: lumbar lordosis, SS: sacral slope, PI: pelvic incidence, PT: pelvic tilt.

The PI and SS had a good correlation in both groups (R = 0.78, p = 0.005 in balanced group; R = 0.63, p = 0.027 in imbalanced group). Considering that the SS is greater than in non achondroplasts, congruent to the sacrum acutum, a relatively greater PI exists, which results in an increase of the pelvic tilt. In patients with a balanced spine, a strong positive correlation between LL and SS was demonstrated (R = 0.94, P < 0.001; Table 2; Fig. 3), indicating that the increased LL compensates for the larger SS in order to maintain sagittal balance. This correlation between LL and SS was only moderately present in the imbalanced patients, and the correlation was not statistically significant (R = 0.45, p = 0.121; Table 3).

**Table 2:** Correlation between spinopelvic parameters in balanced group.

	TLK	PI	SS	SVA	PT	LL	DLL
PI	−0.61†						
SS	−0.48	0.78*					
SVA	0.29	−0.16	−0.16				
PT	−0.58	0.96*	0.57	−0.10			
LL	−0.54†	0.83*	0.94*	−0.24	0.66†		
DLL	−0.17	0.02	0.20	−0.29	−0.12	0.35	
Adapted LL	−0.47	0.82*	0.90*	−0.16	0.68†	0.97*	0.29

*Significant correlations were established at the 0.01 level.

†Significant correlations were established at the 0.05 level.

PI indicates pelvic incidence; SS, sacral slope; PT, pelvic tilt; TLK, thoracolumbar kyphosis; LL, lumbar lordosis; SVA, sagittal vertical axis.

**Table 3:** Correlation between spinopelvic parameters in disbalanced group.

	TLK	PI	SS	SVA	PT	LL	DLL
PI	−0.06						
SS	−0.33	0.63†					
SVA	0.45	0.35	0.00				
PT	0.12	0.90*	0.22	0.43			
LL	−0.63†	−0.08	0.45	−0.42	−0.36		
DLL	0.53	0.15	0.32	0.17	−0.002	−0.05	
Adapted LL	−0.56†	0.16	0.40	−0.63†	−0.44	0.94*	0.02

*Significant correlations were established at the 0.01 level.

†Significant correlations were established at the 0.05 level.

PI indicates pelvic incidence; SS, sacral slope; PT, pelvic tilt; TLK, thoracolumbar kyphosis; LL, lumbar lordosis; SVA, sagittal vertical axis.

**Fig. 4:**
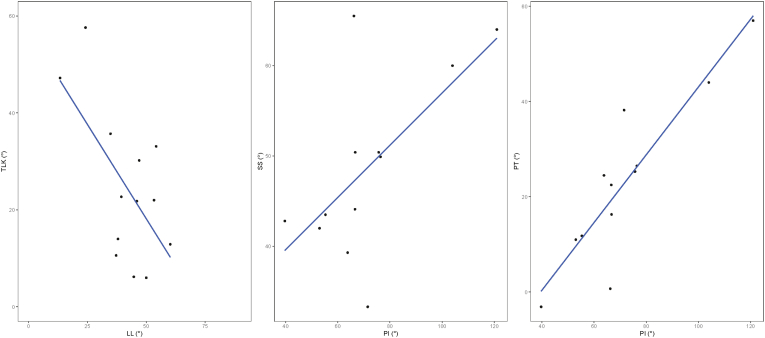
Graphical representation of the relationship between TLK, LL, SS, PI and PT in achondroplast patients with an imbalanced spine. Positive correlations are identified between SS and PI, as well as, PT and PI, and negative correlations are identified between TLK and LL. TLK, thoracolumbar kyphosis, LL, lumbar lordosis, SS, sacral slope, PI, pelvic incidence, PT, pelvic tilt.

In the patients with an imbalanced spine, there was a strong negative correlation between LL and TLK (R = −0.63, p = 0.021, Table 3, Fig. 4), which leads to (partial) compensation for the lacking compensation of the LL for the increased SS. If the adapted LL was considered, this significant negative correlation remained. This correlation was likewise negative in the balanced patients, though with moderate strength (R = −0.54, p = 0.047, Fig. 4) and significance disappeared when the adapted LL was considered.

## 4 Discussion

Sagittal alignment of the spine is the result of a balance in kyphosis and lordosis in various parts of the spine (Diebo et al., 2015; Moal et al., 2014). The achondroplastic spine is, amongst other things, characterized by a sacrum acutum, which is accompanied by an increase in sacral slope. The pelvic incidence is the sum of the sacral slope and the pelvic tilt (Celestre et al., 2018). Extension of the hips is probably limited so that the PT cannot increase beyond a certain magnitude. The reported data presented here illustrate that achondroplasts do not have the capacity to tilt their pelvis to an extent that the sacral slope can be corrected to values comparable to non achondroplasts. To maintain sagittal balance and avoid forward deviation, compensation must occur through lumbar lordosis. However, if the lumbar lordosis is very large, the thoracolumbar junction has to kyphosis in order to avoid backward deviation. This is supported by the data presented in this study, which demonstrated TLK and LL were significantly correlated with each other, in sagittally balanced- and imbalanced patients.

Though SS and TLK are comparable in both balanced and imbalanced patients, the LL is different between the two groups; in imbalanced patients LL is smaller and it fails to correlate to SS. Both SS and TLK find their final shape in the first years of childhood in achondroplasts (Pauli, 2019). The lumbar spine remains flexible and can theoretically adapt its shape to establish a sagittal balanced spine (Abousamra et al., 2019). If however a considerable TLK is present, this adds a constant extra contact force on the anterior column of the lumbar spine in upright position. This prevents the lumbar spine from compensating into lordosis. This is supported by the observation that LL is smaller in imbalanced patients.

In non achondroplasts, the predominant determinant of lumbar lordosis is the distal lordosis of the lumbar spine. Our data illustrate that the DLL is comparable between both groups, although the total LL differs between the groups. Likewise, the correlations between DLL and TLK, as well as between DLL and SS are insignificant, in contrast to the correlations with LL. We hypothesize that the vertical area covered by the lower lumbar spine in achondroplasia is too short to exert the compensation.

The clinical consequence of a imbalanced sagittal spine is not considered in this study. It is imaginable that a continuous imbalance of the spine, in which there is forward deviation, leads to stress in the long muscles of the back. This constant muscle stress can easily lead to chronic back pain (Wang et al., 2022). Adding to that the considerable weight of the relatively heavy skull of the achondroplast, it is even more likely that back pain is the result of this imbalance (Ogura et al., 2019). In the balanced spine patients however, the lumbar lordosis may be so high that the curvature induces a smaller diameter of the spinal canal, on top of the already diminished A-P diameter of the canal. This may induce clinical signs like neurogenic claudication. In future studies the sagittal balance properties of the patients will be combined with clinical data in order to further evaluate the compensatory mechanisms of the achondroplast spine.

It is not known why some Ach patients are able to adapt their lumbar lordosis to maintain sagittal balance and why other patients are unable to do so. It seems reasonable to presume that the aged spine is less tolerant for excessive compensation in lumbar curvature than the younger spine. In order to explore the effect of age on sagittal balance of spine, we would ideally evaluate our data while dichotomizing the population into subjects older and younger than 50 years old. However, the current study population is too small to make a meaningful comparison between age groups.

Our study has several limitations. Firstly, it is a retrospective single-center study with only a limited number of patients that could be evaluated. Secondly, some of the patients were operated on the spine before, which may have influenced their sagittal balance. Thirdly, some of the patients were treated in their childhood to prevent TLK, and these data were not available. Future longitudinal research in which long term follow up of the sagittal balance parameters in achondroplasts from early childhood to adulthood are studied may shed light on this development. Finally, both groups might be not comparable in terms of age and PI. Correction for that however is deemed not feasible considering the limited sample size. With continuing the inclusion and evaluation of patients in our clinic we hope to increase numbers and to be able to analyze this in more depth.

## 5 Conclusion

In achondroplasia sagittal imbalance is a common feature of the spine. It is observed that the imbalance is driving the spine to forward deviation and that this is probably due to an inability to compensate in lumbar lordosis for an increased sacral slope. It is furthermore observed that lumbar lordosis is sufficiently large to compensate for the thoracolumbar kyphosis in balanced spines, but that this compensating mechanism fails in Ach patients with an imbalanced spine.

## Declaration of competing interest

All authors declare that they have no conflicts of interest.
